# Akt Activation Is Responsible for Enhanced Migratory and Invasive Behavior of Arsenic-Transformed Human Bronchial Epithelial Cells

**DOI:** 10.1289/ehp.1104061

**Published:** 2011-09-27

**Authors:** Zhishan Wang, Junling Yang, Theresa Fisher, Hua Xiao, Yiguo Jiang, Chengfeng Yang

**Affiliations:** 1 Department of Physiology, Michigan State University, East Lansing, Michigan, USA; 2 Institute for Chemical Carcinogenesis, State Key Laboratory of Respiratory Diseases, Guangzhou Medical University, Guangzhou, People’s Republic of China; 3 Center for Integrative Toxicology, Michigan State University, East Lansing, Michigan, USA

**Keywords:** Akt, arsenic, EMT, Erk1/2, human bronchial epithelial cells (HBECs), invasion, microRNA 200b (miR-200b), migration, PI3K

## Abstract

Background: Arsenic is one of the most common environmental contaminants. Long-term exposure to arsenic causes human bronchial epithelial cell (HBEC) malignant transformation and lung cancer. However, the mechanism of arsenic lung carcinogenesis is not clear, and the migratory and invasive properties of arsenic-transformed cells (As-transformed cells) have rarely been studied.

Objectives: This study was designed to investigate the migratory and invasive behavior of As-transformed HBECs and the underlying mechanism.

Methods: As-transformed p53^low^HBECs were generated by exposing p53-knockdown HBECs to sodium arsenite (2.5 μM) for 16 weeks. Cell migration was assessed by transwell migration and wound-healing assay. Cell invasion was evaluated using Matrigel-coated transwell chambers. Gene overexpression, small interfering RNA (siRNA) knockdowns, and pharmacological inhibitors were used to determine the potential mechanism responsible for enhanced cell migration and invasion.

Results: Transwell migration and invasion assays revealed that As-transformed p53^low^HBECs were highly migratory and invasive. Akt (also known as protein kinase B) and extracellular signal–regulated protein kinase 1/2 (Erk1/2) were strongly activated in As-transformed p53^low^HBECs. Stable expression of microRNA 200b in As-transformed p53^low^HBECs abolished Akt and Erk1/2 activation and completely suppressed cell migration and invasion. Pharmacological inactivation of Akt but not Erk1/2 significantly decreased cell migration and invasion. Inhibition of Akt reduced the expression of epithelial-to-mesenchymal transition–inducing transcription factors zinc-finger E-box–binding homeobox factor 1 (ZEB1) and ZEB2. siRNA knockdown of ZEB1 and ZEB2 impaired As-transformed p53^low^HBEC migration and invasion.

Conclusions: Akt activation plays a critical role in enabling As-transformed HBEC migration and invasion by promoting ZEB1 and ZEB2 expression.

Arsenic is one of the most common environmental pollutants, occurring naturally in rocks, soil, and water. It can be released into the environment through natural activities or human actions. While human arsenic exposure may occur through environmental, occupational, and medicinal sources, contaminated drinking water is the main source of general population exposure. Arsenic levels in drinking water often exceed many times the World Health Organization’s recommendation of 10 ppb (10 μg/L), affecting tens of millions of people in the United States and many other countries (States et al. 2009; Tapio and Grosche 2006). Although epidemiological studies have consistently shown that long-term arsenic exposure through drinking water is associated with increased risks of skin, lung, bladder, liver, and prostate cancers, the underlying mechanisms have not been elucidated (Benbrahim-Tallaa and Waalkes 2008; Celik et al. 2008; Liu and Waalkes 2008; Tapio and Grosche 2006).

Chronic exposure to arsenic causes malignant transformation of various animal and human cells. For example Zhao et al. (1997) reported that sodium arsenite (0.5 μM) induced transformation of rat liver epithelial TRL 1215 cells, which was accompanied by a morphological change of the cells from epithelioid to fibroblast-like. Subcutaneous xenograft tumors produced by inoculation of arsenic-transformed TRL 1215 cells showed frequent invasion into the subdermal muscle layers and a high proportion of metastases to the lung (Zhao et al. 1997). Achanzar et al. (2002) found that chronic arsenite (5 μM) exposure caused transformation of immortalized human prostate epithelial RWPE-1 cells. When inoculated into the renal capsules of male NCr-nu nude mice, arsenite-transformed RWPE-1 cells produced undifferentiated epithelial tumors that frequently invaded into the surrounding capsular muscle tissue (Achanzar et al. 2002). Our recent study showed that arsenite (2.5 μM) exposure caused depletion of microRNA 200b (one of a large family of small non-coding RNAs; miR-200b), epithelial-to-mesenchymal transition (EMT), and malignant transformation of immortalized human bronchial epithelial cells (HBECs) (Wang et al. 2011). Subcutaneous inoculation of arsenic-transformed HBECs (As-transformed HBECs) produced undifferentiated lung epithelial cell-derived xenograft tumors in Nu-Nu nude mice (Wang et al. 2011; Yang 2011). EMT enables cells to adopt a spindle-shaped fibroblast-like morphology with enhanced migratory capacity and invasiveness, and EMT is believed to play an important role in tumor progression by promoting tumor invasion and metastasis (Lee et al. 2006; Thiery et al. 2009). Therefore, findings from our study along with those of others (Zhao et al. 1997; Achanzar et al. 2002) showing invasive xenograft tumor formation in nude mice resulting from inoculation of As-transformed cells suggest that As-transformed cells may have developed strong migratory and invasive capabilities. However, the migratory and invasive potential of As-transformed cells has rarely been studied.

The present study was designed to investigate the migratory and invasive behavior of As-transformed HBECs and the underlying mechanism. We found that As-transformed HBECs display highly migratory and invasive potentials, and the nude mouse xenograft tumors resulting from subcutaneous inoculation of As-transformed HBECs were capable of invading into surrounding fat tissues and forming tubelike structures. Although both Akt (also known as protein kinase B) and extracellular signal–regulated protein kinase 1/2 (Erk1/2) were strongly activated in As-transformed HBECs, it is Akt, and not Erk1/2, activation that plays a key role in enabling cell migration and invasion. Moreover, we demonstrated that Akt activation drives As-transformed HBEC migration and invasion by promoting the expression of EMT-inducing transcription factors zinc-finger E-box–binding homeobox factor 1 (ZEB1) and ZEB2.

## Materials and Methods

*Cell culture.* Immortalized HBECs with intact p53 expression and function and HBECs with p53 expression stably knocked down (p53^low^HBECs), which were generated from the parental HBECs by expressing a short hairpin RNA targeting p53, were generously provided by J.D. Minna (University of Texas Southwestern Medical Center, Dallas, TX, USA) (Ramirez et al. 2004; Sato et al. 2006; Wang et al. 2011). Both p53-intact HBECs and p53^low^HBECs were cultured in chemically defined serum-free medium (K-SFM; Invitrogen, Carlsbad, CA, USA) supplemented with 20 μg/mL of bovine pituitary extract and 0.8 μg/mL epidermal growth factor (EGF). The cell transformation experiment was previously performed by continuous exposure of HBECs and p53^low^HBECs to arsenic (sodium arsenite, 2.5 μM) for 16 weeks (Wang et al. 2011). Sixteen-week arsenic exposure caused malignant transformation of only p53^low^HBECs and not p53-intact HBECs (Wang et al. 2011). Arsenic-transformed cells (As-transformed p53^low^HBECs) were cultured in K-SFM as above with the same supplements in the absence of arsenic.

*Transwell cell migration and invasion assay.* Control cell and As-transformed cell migration and invasion were quantified by transwell assays using uncoated (8 μm pore size; Corning Costar, Cambridge, MA, USA) or growth factor–reduced Matrigel™-coated filters (8 μm pore size; BD Biosciences, Franklin Lakes, NJ, USA) in 24-well plates, respectively. Briefly, cells were trypsinized and seeded onto the upper chamber of the transwells (5 × 10^4^ cells/well) in supplement-free K-SFM. The lower chamber of the transwells was filled with K-SFM containing 100 ng/mL EGF. The chambers were incubated at 37°C with 5% CO_2_ for 6 hr (migration assay) or 24 hr (invasion assay). At the end of incubation, cells on the upper surface of the filter were removed using a cotton swab. Cells migrating or invading through the filter to the lower surface were fixed with 4% paraformaldehyde for 10 min and stained with 0.1% crystal violet for 5 min. Migrated or invaded cells were viewed and photographed under a phase-contrast microscope and counted in five fields (100× magnification). The fields were randomly chosen from the top, bottom, left, right, and center position of each filter. The person who counted the cells was not aware of which experimental group of cells was being counted. The experiments were performed in triplicate wells and performed two to three times.

*Wound-healing cell migration assay.* To examine the effect of inhibition of phosphoinositide 3-kinase (PI3K), Akt, or Erk1/2 on cell migration, a wound-healing assay was performed. Briefly, As-transformed cells were seeded into 6-cm dishes and allowed to form confluent monolayers. Cell monolayers were scratched using a 200-μL pipette tip to create a wound and washed once with phosphate-buffered saline (PBS); then we added fresh K-SFM culture medium supplemented with 1 μg/mL of the proliferation inhibitor mitomycin C (Sigma, St. Louis, MO, USA), and vehicle control [dimethyl sulfoxide (DMSO); Sigma], 1 μM of the PI3K inhibitor wortmannin (EMD Chemicals USA, Gibbstown, NY, USA), 5 μM of the Akt inhibitor VIII trifluoroacetate salt hydrate (Sigma), or 2.5 μM of the MEK1 [mitogen-activated protein kinase (MAPK)/ERK kinase 1] inhibitor U0126 (EMD Chemicals USA). Wound width was monitored over time by microscopy and photographed immediately after inhibitors were added in (0 hr) and after a 20-hr incubation. Wortmannin (1 μM) was added in again after 10 hr of incubation. The experiments were performed in triplicate dishes and repeated two to three times.

*Generation of cells stably expressing miR-200b.* Establishment of stable expression of miR-200b in As-transformed p53^low^HBEC cells was described in detail previously (Wang et al. 2011).

*Western blot analysis.* Cells were lysed using Tris-sodium dodecyl sulfate (SDS) as described by Yang et al. (2006) and subjected to SDS–polyacrylamide gel electrophoresis (10–50 μg of protein/lane). The following primary antibodies were used: anti-phosphorylated-Akt (Ser473), anti-phosphorylated-Akt (Thr308), anti-total-Akt, anti-phosphorylated-Erk1/2, anti-total-Erk1/2, anti-phosphorylated-p38, anti-phosphorylated-JNK (c-jun *N*-terminal kinase) (Cell Signaling Technology, Beverly, MA, USA); anti-ZEB1 (Santa Cruz Biotechnology, Inc., Santa Cruz, CA, USA); anti-ZEB2 (Novus Biologicals, Littleton, CO, USA); and anti-β-actin (Sigma).

*ZEB1 and ZEB2 RNA interference.* Negative control small interfering RNA (siRNA) and ON-TARGETplus SMARTpool siRNA for ZEB1 and ZEB2 were obtained from Thermo Scientific Dharmacon (Lafayette, CO, USA). To investigate the role of ZEB1 and ZEB2 in As-transformed cell migration and invasion, siRNA duplexes (100 nM) were transfected into cells using Lipofectamine 2000 (Invitrogen) in serum-free medium as described previously (Wang et al. 2011). For simultaneous knockdown of both ZEB1 and ZEB2, 50 nM of ZEB1 and 50 nM of ZEB2 siRNA duplexes were used. Forty-eight hours after transfection, cells were collected for transwell migration/invasion assay and Western blot analysis as described above.

*Thyroid transcription factor 1 immunofluorescence staining.* Nude mouse subcutaneous xenograft tumor tissue sections (5 μm) from our previous study were prepared and subjected to hematoxylin and eosin and immunofluorescence staining as described previously (Wang et al. 2011; Yang 2011). Anti-thyroid transcription factor 1 (anti-TTF-1; sc-13040) primary antibody was obtained from Santa Cruz Biotechnology. Slides were counterstained with 4´,6-diamidino-2-phenylindole (DAPI). The stained sections were visualized with a Nikon Eclipse TE2000-U fluorescence microscope (Nikon, Inc., Melville, NY, USA). The captured red fluorescent images (TTF-1–positive staining) were overlaid with the blue fluorescent images (nucleus DAPI staining) using MetaMorph software (Molecular Devices Corp., Downington, PA, USA).

*Statistical analysis.* The statistical analyses for the significance of differences in numerical data (means ± SDs) were performed using two-tailed *t*-tests for comparison of two data sets or one-way analysis of variance for multiple data sets. A *p*-value of < 0.05 was considered statistically significant.

## Results

**Figure 1:**
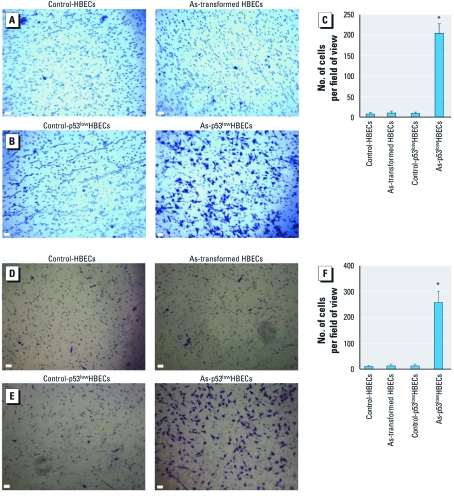
As-transformed p53^low^HBECs are highly migratory and invasive. (*A*, *B*, *D*, and *E*) Representative images of transwell cell migration (*A* and *B*) and invasion (*D* and *E*) assays. For details, see “Materials and Methods.” Bars = 100 μm. (*C* and *F*) Quantification of transwell cell migration (*C*) and invasion (*F*) assays (means ± SDs, *n* = 3). Similar results were obtained in two additional experiments. *p < 0.05, compared to vehicle control–exposed cells or As-transformed HBECs.

*As-transformed p53^low^HBECs exhibit high migratory and invasive potentials.* We recently found that subcutaneous inoculation of As-transformed p53^low^HBECs into nude mice produced undifferentiated lung epithelial-derived xenograft tumors (Wang et al. 2011; Yang 2011). Histological analysis revealed that the mouse xenograft tumors were capable of invading into surrounding fat tissues and forming tubelike structures [see Supplemental Material, Figure 1 (http://dx.doi.org/10.1289/ehp.1104061)]. Extensive positive staining of TTF-1, which is present in the epithelium of the lung and used as a marker of human primary lung tumors (Reis-Filho et al. 2000), was detected in xenogaft tumors (Yang 2011) and their surrounding tubelike structures (Supplemental Material, Figure 2). These results suggest that mouse xenograft tumors resulting from subcutaneous inoculation of As-transformed HBECs display a strong invasive property. This finding is consistent with previous studies showing that inoculation of rat liver epithelial As-transformed cells or human prostate epithelial cells grew invasive tumors in immunocompromised mice (Achanzar et al. 2002; Zhao et al. 1997). However, the migratory and invasive behavior of As-transformed cells has been rarely studied. Because EMT enables cell to move and invade (Lee et al. 2006; Thiery et al. 2009), we examined the migratory and invasive properties of As-transformed HBECs that underwent EMT. Control HBECs and p53^low^HBECs displayed weak migratory and invasive capability as determined by transwell migration and invasion assays (Figure 1A,B,D,E). In striking contrast, As-transformed p53^low^HBECs showed strong migratory capacity (Figure 1B,C) and invasive potential (Figure 1E,F). Consistent with our previous finding that HBECs exposed to arsenic for 16 weeks did not undergo EMT and malignant transformation (Wang et al. 2011), As-exposed HBECs exhibited only weak migration and invasion comparable to that of control HBECs. These results along with our recent findings indicate that As-transformed p53^low^HBECs are highly migratory and invasive.

**Figure 2:**
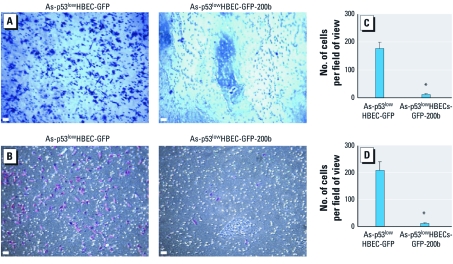
Stable reexpression of miR-200b completely inhibits As-transformed HBEC migration and invasion. (*A* and *B*) Representative images of transwell cell migration (*A*) and invasion (*B*) assays, as described in “Materials and Methods.” Bars = 100 μm. (*C* and *D*) Quantification of transwell cell migration (*C*) and invasion (*D*) assays (means ± SD, *n* = 3). Similar results were obtained in two additional experiments. *p < 0.05, compared with As-transformed p53^low^HBEC-GFP cells.

*Stable reexpresson of miR-200b completely inhibits As-transformed HBEC migration and invasion.* Stable reexpresson of miR-200b in As-transformed p53^low^HBECs reversed their transformed phenotypes, as evidenced by inhibition of colony formation in soft agar and prevention of xenograft tumor formation in nude mice (Wang et al. 2011). We then wanted to determine whether stably reexpressing miR-200b has an effect on As-transformed p53^low^HBEC migratory and invasive behavior. Green fluorescent protein (GFP) vector control cells displayed similar migratory (Figure 2A,C) and invasive potentials (Figure 2B,D), comparable to those of As-transformed p53^low^HBECs. However, cells stably expressing miR-200b lost the migratory and invasive capability, exhibiting only weak migration and invasion similar to that of nontransformed cells (compare Figures 1 and 2). These results indicate that stably reexpressing miR-200b completely inhibits As-transformed HBEC migration and invasion.

**Figure 3:**
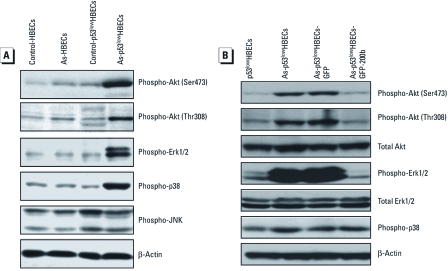
Akt, Erk1/2, and p38 MAPK are highly activated in As-transformed HBECs, and stably reexpressing miR-200b abolishes Akt and Erk1/2 activation. (*A*) Akt, Erk1/2, and p38 MAPK are highly activated only in As-transformed p53^low^HBECs. (*B*) Stable reexpression of miR-200b abolishes Akt and Erk1/2 activation but has no effect on p38 MAPK activation in As-transformed p53^low^HBECs. Similar results were obtained in two additional experiments.

*MiR-200b abolishes Akt and Erk1/2 activation in As-transformed HBECs.* Previous studies have shown that arsenic exposure can activate Akt, Erk1/2, p38, and JNK pathways and that Akt and Erk1/2 activation plays important roles in arsenic-induced cell transformation (Eblin et al. 2007; Huang et al. 1999). However, it remains to be determined whether Akt, Erk1/2, or another signaling pathway activation is responsible for promoting As-transformed cell migration and invasion. We first examined the activation status of Akt, Erk1/2, p38, and JNK in control cells, As-exposed HBECs, and As-transformed p53^low^HBECs. Strikingly, Akt, Erk1/2, and p38 MAPK were highly phosphorylated in As-transformed p53^low^HBECs (Figure 3A), whereas no dramatic differences of JNK phosphorylation were detected between control cells and As-exposed HBECs. Since stably reexpressing miR-200b completely inhibited As-transformed p53^low^HBEC migration and invasion, we next determined the effect of miR-200b on Akt, Erk1/2, and p38 activation status in As-transformed p53^low^HBECs. Stable reexpression of miR-200b abolished Akt and Erk1/2 phosphorylation but had no effect on p38 phosphorylation (Figure 3B). These results suggest that activation of Akt and Erk1/2, but not p38, may contribute to enhanced migratory and invasive behavior of As-transformed p53^low^HBECs.

**Figure 4:**
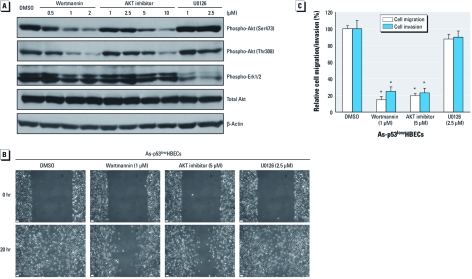
Inhibition of Akt but not Erk1/2 suppresses As-transformed HBEC migration and invasion. (*A*) Inhibition of Akt and Erk1/2 determined by Western blot analysis. (*B*) Inhibition of Akt but not Erk1/2 impairs As-transformed p53^low^HBEC migration determined by the wound-healing assay. Bars = 100 μm. (*C*) Inhibition of Akt but not Erk1/2 suppresses As-transformed p53^low^HBEC migration and invasion as determined by transwell migration/invasion assays (means ± SDs, *n* = 3). Similar results were obtained in two additional experiments. *p < 0.05, compared with vehicle control–treated cells.

*Akt but not Erk1/2 activation enables As-transformed HBEC migration and invasion.* To determine the potential role of Akt and/or Erk1/2 activation in cell migration and invasion, As-transformed p53^low^HBECs were treated with a PI3K inhibitor (wortmannin), an Akt inhibitor, or an MEK1 inhibitor (U0126) that blocks Erk1/2 activation. As expected, wortmannin and the Akt inhibitor efficiently and dose-dependently inhibited Akt phosphorylation with no significant effect on Erk1/2 phosphorylation, whereas U0126 efficiently suppressed Erk1/2 phosphorylation with no effect on Akt phosphorylation (Figure 4A). The wound-healing assay revealed that wortmannin and Akt inhibitor treatment significantly reduced wound closure, indicating significant inhibition of cell migration (Figure 4B). In contrast, inhibition of Erk1/2 by U0126 showed no significant effect on wound closure, indicating no inhibition of cell migration. These effects were further confirmed by using transwell cell migration and invasion assays (Figure 4C). Treatment with wortmannin and Akt inhibitor significantly reduced cell migration and invasion by 80–85% and 75–77%, respectively. However, treatment with U0126 only slightly reduced cell migration and invasion, by 12% and 10%, respectively (Figure 4C). These results indicate that it is mainly the Akt activation that drives As-transformed HBEC migration and invasion.

**Figure 5:**
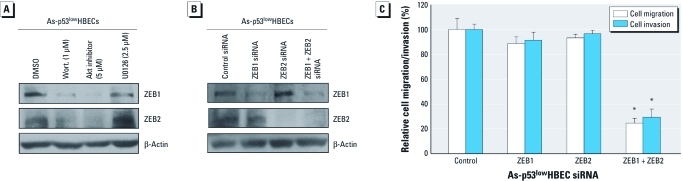
Inhibition of Akt decreases the expression of ZEB1 and ZEB2, and siRNA knockdown of ZEB1 and ZEB2 reduces As-transformed p53^low^HBEC migration and invasion. (*A*) Inhibition of Akt but not Erk1/2 decreases the expression of ZEB1 and ZEB2. Wort., wortmannin. (*B*) siRNA knockdown of the expression of ZEB1 and ZEB2. (*C*) siRNA knockdown of ZEB1 and ZEB2 reduces As-transformed p53^low^HBEC migration and invasion (means ± SDs, *n* = 3). Similar results were obtained in two additional experiments. *p < 0.05, compared with control siRNA-transfected cells.

*Akt activation enables As-transformed HBEC migration and invasion via promoting ZEB1 and ZEB2 expression.* We investigated how Akt activation promotes As-transformed p53^low^HBEC migration and invasion. We previously showed that arsenic exposure caused EMT through inducing the expression of ZEB1 and ZEB2 (Wang et al. 2011). ZEB1 and ZEB2 are EMT-inducing transcription factors capable of promoting cell migration and invasion (Kim et al. 2011). We then examined whether inhibition of Akt activation has an effect on ZEB1 and ZEB2 expression. Figure 5A shows that treatment with wortmannin (1 μM) and an Akt inhibitor (5 μM), which efficiently reduced Akt phosphorylation and cell migration/invasion and greatly decreased the protein levels of ZEB1 and ZEB2. As expected with the lack of significant effect on cell migration and invasion by inhibition of Erk1/2, treatment with U0126 (2.5 μM) also did not reduce the protein levels of ZEB1 and ZEB2.

To determine the role of ZEB1 and ZEB2 in As-transformed p53^low^HBEC migration and invasion, the siRNA approach was used to knock down the expression of ZEB1 and ZEB2. Figure 5B shows efficient knockdown of ZEB1 and ZEB2 using ON-TARGETplus SMARTpool siRNA for ZEB1 and ZEB2 (Wang et al. 2011) as determined by Western blot. Knockdown of either ZEB1 or ZEB2 did not have a significant effect on cell migration and invasion (Figure 5C). However, simultaneous knockdown of both ZEB1 and ZEB2 significantly reduced cell migration and invasion, suggesting that there is an overlap function between ZEB1 and ZEB2 in promoting cell migration and invasion. Together, these results indicate that Akt activation drives As-transformed HBEC migration and invasion mainly through promoting ZEB1 and ZEB2 expression.

## Discussion

Arsenic exposure represents a major environmental health concernin causing cancers and other diseases. The mechanism of arsenic carcinogenesis, particularly of arsenic lung carcinogenesis, has not been elucidated. It is generally accepted that nongenotoxic modifications that lead to aberrant gene expression may play key roles in arsenic carcinogenesis (Arita and Costa 2009; Hernandez et al. 2009; Ren et al. 2011; Yang and Frenkel 2002). Indeed, studies have shown that abnormal cell signaling and gene expression are critically involved in various animal and human cell transformation process by arsenic exposure (Eblin et al. 2007; Huang et al. 1999; Ouyang et al. 2008; Wang et al. 2011). In the present study, we provided additional novel evidence suggesting that abnormal cell signaling and gene expression may play a crucial role in arsenic lung carcinogenesis by promoting As-transformed HBEC migration and invasion.

Arsenic-induced cell transformation has been reported to be accompanied by cellular morphological changes from epithelioid to fibroblast-like (Chang et al. 2010; Zhao et al. 1997). When inoculated into nude mice, rat liver epithelial As-transformed cells and human prostate epithelial cells produced invasive xenograft tumors (Achanzar et al. 2002; Zhao et al. 1997). In addition, Wnek et al. (2010) found that the changes of expression of two biomarkers (cyclooxygenase-2 and gene promoter “deleted in bladder cancer 1”) of invasive bladder cancers were associated with monomethylarsonous acid–induced malignant transformation of human urothelial cells (UROtsa). We found that subcutaneous inoculation of As-transformed HBECs into nude mice produced undifferentiated lung epithelial-derived xenograft tumors (Wang et al. 2011; Yang 2011), which were capable of invading into the surrounding fat tissues and forming tubelike structures. Furthermore, our and other recent studies (Li et al. 2011; Wang et al. 2011) demonstrated that arsenic exposure induced EMT of rat lung epithelial cells and HBECs, as evidenced by the appearance of spindle-shaped morphology, loss of epithelial cell marker E-cadherin, and acquisition of mesenchymal cell markers vimentin and N-cadherin. EMT occurs as part of normal embryonic development, enabling cells to migrate; EMT is now viewed as an important step in tumor invasion and metastasis (Kalluri and Weinberg 2009). Together, these findings indicate that investigating the migratory and invasive properties of As-transformed cells is essential. The results presented here demonstrate that As-transformed HBECs obtained strong motile capacity and invasive potential. However, it is interesting to note that short-term (36 hr) sodium arsenite treatment blocked chicken cardiac endothelial-to-mesenchymal transition (Lencinas et al. 2010), suggesting that arsenic may have differential effects on epithelial- versus endothelial-to-mesenchymal transition.

Previous studies have shown that acute and chronic arsenic exposure activated Akt and Erk1/2 (Eblin et al. 2007; Simeonova et al. 2002). Akt and Erk1/2 activity were critically involved in arsenic-induced human and mouse cell transformation (Eblin et al. 2007; Huang et al. 1999). Ouyang et al. (2008) further determined that Akt activation promoted human keratinocyte transformation by arsenic by increasing cyclin D1 expression. Collectively, these earlier studies showed critical roles for Akt and Erk1/2 activation in cell transformation by arsenic. Nevertheless, whether Akt or Erk1/2 activation or other abnormally activated signaling pathways play a role in migratory and invasive behavior of As-transformed cells had not been investigated. By overexpressing miR-200b, applying pharmacological inhibitors to inactivate Akt or Erk1/2, and siRNA knockdown of the expression of ZEB1 and ZEB2, we established that it is the activation of Akt, and not Erk1/2, that enables As-transformed HBECs to migrate and invade. These findings along with others mentioned above suggest that Akt activation not only plays important roles in the early-stages of arsenic carcinogenesis by promoting cell transformation, but also may play a key role in the late stages of arsenic carcinogenesis by driving As-transformed cell migration and invasion. This is consistent with previous studies showing hyperactivation of Akt in the majority of human lung cancers and inhibition of Akt suppressing metastatic potential of human lung cancer cells (Akca et al. 2011; Altomare and Testa 2005; Ogata et al. 2011).

Arsenic exposure can activate Akt in a PI3K-depedent manner in human urothelial cells and keratinocytes (Eblin et al. 2007; Ouyang et al. 2008). In addition, Zhang et al. (2011) recently found that down-regulation of protein phosphatase 2A and pleckstrin homology domain leucine-rich repeat protein phosphatase 2 contribute to Akt activation in arsenite-treated mouse epidermal JB6 Cl41 cells. These findings indicate that different mechanisms of Akt activation by arsenic exposure exist in different kinds of cells. In the present study, we found that Akt activation in As-transformed HBECs depended on PI3K activity as inhibition of PI3K diminished Akt phosphorylation at both serine 473 and threonine 308 sites. Moreover, inactivation of Akt by treatment with a PI3K inhibitor or an Akt inhibitor similarly and significantly reduced the expression of ZEB1 and ZEB2, cell migration, and invasion.

ZEB1 and ZEB2 are EMT-inducing transcription factors (Peinado et al. 2007). Our recent study indicated that arsenic exposure caused EMT of p53^low^HBECs by inducing the expression of ZEB1 and ZEB2 without affecting the expression of other EMT-inducing transcription factors (Wang et al. 2011). While EMT is generally considered a late event in cancer progression, our and other recent findings provide evidence that EMT may also play a role in arsenic-caused or tobacco-carcinogen–caused HBEC transformation, the initial step of carcinogenesis (Tellez et al. 2011; Wang et al. 2011). Although we demonstrated an important role for Akt activation in promoting As-transformed HBEC migration and invasion, it remains to be determined whether Akt is activated during the early stage of HBEC transformation and whether Akt activation is required for HBEC transformation by arsenic. Because Akt can be activated in human urothelial cells and keratinocytes during short- and long-term arsenic exposure (Eblin et al. 2007; Ouyang et al. 2008), further studies are warranted to investigate Akt activation status in HBECs during early arsenic exposure and whether Akt activation contributes to HBEC transformation by arsenic. Findings from this and future studies will help establish Akt as a key molecular target for the prevention and treatment of human lung cancer resulting from arsenic exposure.

## Conclusions

The present study is the first to focus on characterizing As-transformed cell migratory and invasive behavior and the underlying mechanism. We demonstrated that As-transformed HBECs have strong migratory and invasive capability. While both Akt and Erk1/2 are highly activated in As-transformed HBECs, it is mainly the activation of Akt, and not Erk/1/2, that enables As-transformed HBEC migration and invasion. Further, Akt activation drives cell migration and invasion through promoting the expression of EMT-inducing transcription factors ZEB1 and ZEB2. Given the critical role of Akt, ZEB1, and ZEB2 in cancer progression (Altomare and Testa 2005; LoPiccolo et al. 2008; Peinado et al. 2007), the findings from this study provide additional novel evidence that Akt activation may play an important role in arsenic lung carcinogenesis by promoting As-transformed HBEC migration and invasion.

## Supplemental Material

(160 KB) PDF
