# Supplementary material for: Akt Activation Is Responsible for Enhanced Migratory and Invasive Behavior of Arsenic-Transformed Human Bronchial Epithelial Cells
Source: Environ Health Perspect. 2011 Sep 27;120(1):92–7. doi: 10.1289/ehp.1104061 (PMC3261952; doi:10.1289/ehp.1104061)
Supplement: (160 KB) PDF [file ehp.1104061.s001.pdf]

Supplemental Materials

Akt Activation is Responsible for Enhanced Migratory and Invasive Behavior of Arsenic-Transformed Human Bronchial Epithelial Cells

Zhishan Wang, Junling Yang, Theresa Fisher, Hua Xiao, Yiguo Jiang,

Chengfeng Yang

Table of Contents

Supplementary Figure 1……………………………………………..Page 2

Supplementary Figure 2…………………………………………… Page 3

Reference…………………………………………………………..Page 4

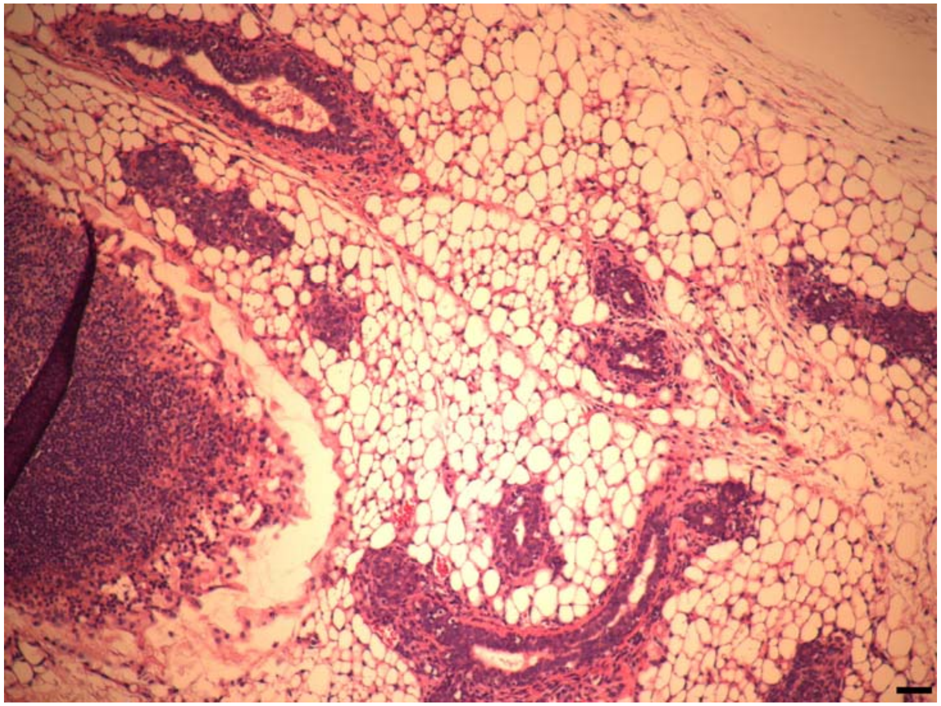

Supplementary Figure 1. Representative H & E staining of mouse xenograft tumor and surrounding fat tissues. The mouse xenograft tumor was produced from subcutaneous inoculation of arsenic-transformed HBECs (As-p53^{low}HBEC-GFP) (Wang et al. 2011) and H & E staining was carried out as described in Materials and Methods. Scale bar: 100 µm.

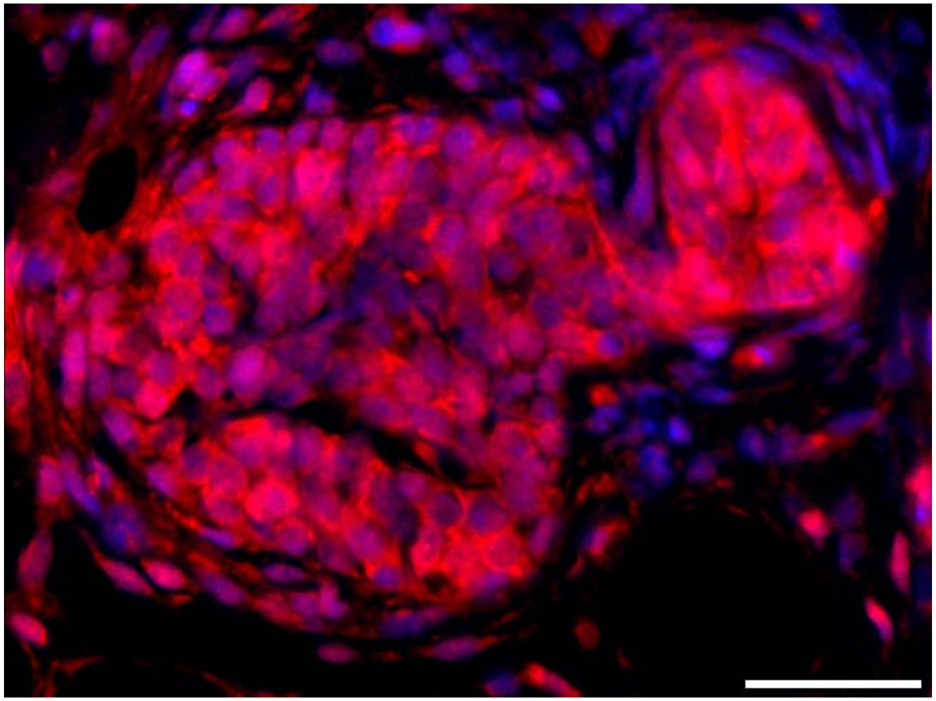

Supplementary Figure 2. Representative immunofluorescence staining of anti-thyroid transcription factor 1 (TTF-1) in mouse xenograft tumor surrounding tube-like structure. Tissue sections from nude mouse xenograft tumors produced by subcutaneous injection of arsenic-transformed HBECs (As-p53^{low}HBEC-GFP) were stained with anti-TTF1 as described in Materials and Methods. Nuclei were counterstained with 4',6-diamidino-2-phenylindole (DAPI) (blue). The overlaid fluorescence image was made from anti-TTF1 staining (red color) and nucleus DAPI staining (blue color). Scale bar: 100 µm.

Reference

Wang Z, Zhao Y, Smith E, Goodall GJ, Drew PA, Brabletz T, et al. 2011. Reversal and Prevention of Arsenic-Induced Human Bronchial Epithelial Cell Malignant Transformation by microRNA-200b. Toxicol Sci 121(1):110-122.
